# Supplementary material for: Impact of Mycoplasma ovipneumoniae on juvenile bighorn sheep (Ovis canadensis) survival in the northern Basin and Range ecosystem
Source: PeerJ. 2021 Jan 19;9:e10710. doi: 10.7717/peerj.10710 (PMC7821761; doi:10.7717/peerj.10710)
Supplement: Supplemental Information 2 [file peerj-09-10710-s002.docx]

Supplementary Material

**Supplementary Tables**

**Table S1.** Translocation histories of bighorn sheep populations included in this study. Details include the population code (Pop) where the bighorn sheep were established or translocated to, translocation type (Trans_type), when the translocation took place (Year), the number of individuals (# ind.) translocated, the source population (Source pop.), the source state or province (S-State) population, the destination population (Destination pop.), and the destination state (D-State).

| **Pop** | **Trans_type** | **Year** | **# individuals** | **Source pop.** | **S-State** | **Destination pop.** | **D-State** |
| --- | --- | --- | --- | --- | --- | --- | --- |
| Bowden Hills | Colonization | unknown | ? | Rattlesnake | OR | Bowden Hills | OR |
| Blue Mountain | Colonization | ~1990s | ? | Trout Ck. | OR | Blue Mountain | OR |
| Calicos | Import | 1985 | 20 | Williams Lake | BC | Pine Forest | NV |
|  | Import | 1988 | 18 | Williams Lake | BC | Pine Forest | NV |
|  | In jurisdiction | 2011 | 25 | Pine Forest | NV | Calico Mtn. | NV |
| Eight Mile | Import | 1978 | 12 | Penticton | BC | Eight Mile | NV |
|  | In jurisdiction | 2014 | 3 | Pine Forest | NV | Three Mile Ck. | NV |
| Martin Creek***** | Import | 1984 | 13 | Hart Mtn. | OR | Jackson Mtn. | NV |
|  | Import | 1985 | 20 | Williams Lake | BC | Pine Forest | NV |
|  | Import | 1986 | 2 | E fork of Owyhee Riv. | ID | Jackson Mtn. | NV |
|  | Import | 1987 | 15 | Lower Owyhee | OR | Jackson Mtn. | NV |
|  | Import | 1988 | 18 | Williams Lake | BC | Pine Forest | NV |
|  | Import | 1989 | 18 | Kamloops | BC | High Rock/Calicos | NV |
|  | In jurisdiction | 1998 | 12 | Jackson Mtn. | NV | Hinkey | NV |
|  | In jurisdiction | 1999 | 12 | High Rock/Calicos | NV | Pine Forest | NV |
|  | In jurisdiction | 2006 | 21 | Montana Mts. | NV | Martin Ck. | NV |
|  | In jurisdiction | 2011 | 27 | Pine Forest | NV | Martin Ck. | NV |
| Rattlesnake | Import | 1954 | 20 |  | BC | Hart Mtn. | OR |
|  | In jurisdiction | 1992 | 19 | Hart Mtn. | OR | Rattlesnake Ck. | OR |
| Sawtooth | Import | 1989 | 20 | Penticton | BC | Sawtooth | NV |
| Trout Creeks – east | Import | 1954 | 20 |  | BC | Hart Mtn. | OR |
|  | In jurisdiction | 1987 | 27 | Hart Mtn. | OR | Trout Creek Mtn. | OR |
| Trout Creeks – south | Import | 1954 | 20 |  | BC | Hart Mtn. | OR |
|  | In jurisdiction | 1990 | 14 | Hart Mtn. | OR | Trout Creek Mtn. | OR |
| Trout Creeks – west | Import | 1954 | 20 |  | BC | Hart Mtn. | OR |
|  | In jurisdiction | 1990 | 19 | Hart Mtn. | OR | Trout Creek Mtn. | OR |
| Ten Mile | Import | 1954 | 20 | Williams Lake | BC | Hart Mtn. | OR |
|  | In jurisdiction | 1960 | 4 | Hart Mtn. | OR | Steens Mtn. | OR |
|  | In jurisdiction | 1961 | 7 | Hart Mtn. | OR | Steens Mtn. | OR |
|  | In jurisdiction | 1989 | 17 | Hart Mtn. | OR | Steens Mtn. | OR |
|  | In jurisdiction | 1992 | 15 | Hart Mtn. | OR | Steens Mtn. | OR |
|  | In jurisdiction | 1993 | 17 | Hart Mtn. | OR | Steens Mtn. | OR |
|  | In jurisdiction | 1993 | 18 | Hart Mtn. | OR | Steens Mtn. | OR |
|  | In jurisdiction | 1993 | 15 | Steens Mtn. | OR | Ten Mile Rim | OR |
| Upper Owyhee***** | Import | 1954 | 20 |  | BC | Hart Mtn. | OR |
|  | In jurisdiction | 1960 | 4 | Hart Mtn. | OR | Steens Mtn. | OR |
|  | In jurisdiction | 1961 | 7 | Hart Mtn. | OR | Steens Mtn. | OR |
|  | In jurisdiction | 1965 | 17 | Hart Mtn. | OR | Lower Owyhee | OR |
|  | In jurisdiction | 1983 | 21 | Hart Mtn. | OR | Lower Owyhee | OR |
|  | In jurisdiction | 1983 | 14 | Hart Mtn. | OR | Upper Owyhee | OR |
|  | In jurisdiction | 1987 | 15 | Hart Mtn. | OR | Lower Owyhee | OR |
|  | In jurisdiction | 1987 | 16 | Hart Mtn. | OR | Lower Owyhee | OR |
|  | In jurisdiction | 1989 | 17 | Hart Mtn. | OR | Steens Mtn. | OR |
|  | In jurisdiction | 1992 | 15 | Hart Mtn. | OR | Steens Mtn. | OR |
|  | In jurisdiction | 1993 | 17 | Hart Mtn. | OR | Steens Mtn. | OR |
|  | In jurisdiction | 1993 | 18 | Hart Mtn. | OR | Steens Mtn. | OR |
|  | In jurisdiction | 1993 | 36 | Steens Mtn. | OR | Upper Owyhee | OR |
|  | In jurisdiction | 1994 | 21 | Lower Owyhee | OR | Upper Owyhee | OR |
|  | In jurisdiction | 1995 | 17 | Hart Mtn. | OR | Upper Owyhee | OR |
|  | In jurisdiction | 2007 | 21 | Philippi Canyon | OR | Upper Owyhee | OR |

*****Indicates incomplete history

**Table S2.** Microsatellite loci used for analysis of bighorn sheep (*Ovis canadensis*) population genetic diversity in southeastern Oregon and Nevada, with allele sizes ranges observed in this study, fluorescent dye labels used, primer concentrations, and pre-PCR multiplex combination employed.

|  |  |  |  | **Primer** |  |
| --- | --- | --- | --- | --- | --- |
|  |  | **Allele size** | **Dye** | **Concentration** |  |
| **Locus** | **Reference** | **(bp)** | **Label** | **(μM)** | **Panel** |
| AE129 | Penty et al*.*, 1993 | 166–177 | Vic | 0.25 | 1 |
| AE16 | Penty et al., 1993 | 84–100 | Fam | 0.20 | 3 |
| BL4 | Smith et al., 1997 | 158–162 | Ned | 0.30 | 2 |
| FCB11 | Buchanan & Crawford, 1993 | 125–131 | Vic | 0.20 | 3 |
| FCB193 | Buchanan & Crawford, 1993 | 105–119 | Pet | 0.25 | 1 |
| FCB266 | Buchanan & Crawford, 1993 | 89–101 | Vic | 0.20 | 3 |
| FCB304 | Buchanan & Crawford, 1993 | 142–150 | Pet | 0.20 | 3 |
| HH62 | Ede et al., 1994 | 102–130 | Fam | 0.15 | 1 |
| JMP29 | Crawford et al., 1995 | 121–133 | Ned | 0.20 | 3 |
| MAF33 | Buchanan & Crawford, 1992b | 122–126 | Vic | 0.25 | 1 |
| MAF36 | Swarbrick et al., 1991 | 87–99 | Vic | 0.15 | 2 |
| MAF48 | Buchanan, Swarbrick & Crawford, 1991 | 122–126 | Ned | 0.20 | 1 |
| MAF65 | Buchanan, Swarbrick & Crawford, 1992 | 118–138 | Fam | 0.20 | 2 |
| MAF209 | Buchanan & Crawford, 1992a | 110–122 | Pet | 0.20 | 2 |
| TCRBV62 | Crawford et al., 1995 | 171–175 | Fam | 0.25 | 3 |
| TGLA387 | Georges & Massey 1992 | 143–151 | Pet | 0.35 | 1 |

**Literature Cited (Table S2)**

Buchanan FC, Crawford AM. 1992a. Ovine dinucleotide repeat polymorphism at the MAF209 locus. *Animal Genetics* 23:183-183.

Buchanan FC, Crawford AM. 1992b. Ovine dinucleotide repeat polymorphism at the MAF33 locus. *Animal Genetics* 23:186-186.

Buchanan FC, Crawford AM. 1993. Ovine microsatellites at the OarFCB11, OarFCB128, OarFCB193, OarFCB266, and OarFCB304 loci. *Animal Genetics* 24:145-145.

Buchanan FC, Swarbrick PA, Crawford AM. 1991. Ovine dinucleotide repeat polymorphism at the MAF48 locus. *Animal Genetics* 22: 379-380.

Buchanan FC, Swarbrick PA, Crawford AM. 1992. Ovine dinucleotide repeat polymorphism at the MAF65 locus. *Animal Genetics* 23:85-85.

Crawford AM, Dodds KG, Ede AJ, Montgomery GW, Garmomway HG, Beattie E, Davies K, Maddox JF, Broom JE, Buitkamp J, Matthew ME, Hulme DJ, Beh KJ, Beattie CW. 1995. An Autosomal Genetic Linkage Map of the Sheep Genome. *Genetics* 140:703–724.

Ede, AJ, Peirson CA, Henry H, Crawford AM. 1994. Ovine microsatellites at the OARAE64, OARHH22, OARHH56, OARHH62 and OARVH4 loci. *Animal Genetics* 25:51-51.

Georges M, Massey J. 1992. Polymorphic DNA markers in Bovidae. Canada Patent W092/13102.

Penty JM, Henry HM, Ede AJ, Crawford AM. 1993. Ovine microsatellites at the OarAE16,

OarAE54, OarAE57, OarAE119 and OarAE129 loci. *Animal Genetics* 24:219-219.

DOI:10.1111/j.1365-2052.1993.tb00295.x

Smith TPL, Lopez-Corrales N, Grosz MD, Beattie CW, Kappes SM. 1997. Anchoring of bovine chromosomes 4, 6, 7, 10, and 14 linkage group telomeric ends via FISH analysis of lambda clones. *Mammalian Genome* 8:333–336. DOI: 10.1007/s003359900434.

Swarbrick PA, Buchanan FC, Crawford AM. 1991. Ovine dinucleotide repeat polymorphism at the MAF36 locus. *Animal Genetics* 22:377-377.

**Table S3.** Breakdown of *Mycoplasma ovipneumoniae* test results for all female bighorn sheep captured and collared between 2016 and 2018 in populations (n = 13) across southeastern Oregon and northern Nevada. Tests included a PCR used to detect active infections of bighorn sheep to *M. ovipneumoniae* and an cELISA test, used to detect previous exposure to *M. ovipneumoniae* + indicates positive cases; - indicates negative cases; “indeterminate” indicates indeterminate; “unknown” indicates individuals for which there were no samples, and “recaptures” indicates recaptured individuals.

|  |  |  |  |  | ***M. ovipneumoniae* status** | | | | | | | |
| --- | --- | --- | --- | --- | --- | --- | --- | --- | --- | --- | --- | --- |
|  |  |  |  | **PCR** | | | |  | **cELISA** | | | |
| **Population** | **Year** | **n** | **recaptures** | **+** | **-** | **indeterminate** | **unknown** |  | **+** | **-** | **indeterminate** | **unknown** |
| Bowden Hills | 2018 | 3 | - | 0 | 2 | 1 | 0 |  | 3 | 0 | 0 | 0 |
| Blue Mountain | 2016 | 3 | - | 0 | 3 | 0 | 0 |  | 0 | 3 | 0 | 0 |
| Blue Mountain | 2017 | 2 | - | 0 | 2 | 0 | 0 |  | 0 | 2 | 0 | 0 |
| Calicos | 2017 | 3 | - | 0 | 3 | 0 | 0 |  | 0 | 0 | 0 | 3 |
| Calicos | 2018 | 5 | 1 | 0 | 5 | 0 | 0 |  | 3 | 1 | 1 | 0 |
| Eight Mile | 2017 | 4 | - | 1 | 3 | 0 | 0 |  | 0 | 0 | 0 | 4 |
| Eight Mile | 2018 | 5 | 3 | 1 | 4 | 0 | 0 |  | 3 | 2 | 0 | 0 |
| Martin Creek | 2017 | 4 | - | 0 | 4 | 0 | 0 |  | 0 | 0 | 0 | 4 |
| Martin Creek | 2018 | 5 | 3 | 0 | 5 | 0 | 0 |  | 4 | 1 | 0 | 0 |
| Rattlesnake | 2016 | 10 | - | 1 | 9 | 0 | 0 |  | 6 | 1 | 3 | 0 |
| Rattlesnake | 2017 | 11 | - | 0 | 11 | 0 | 0 |  | 7 | 1 | 3 | 0 |
| Sawtooth | 2017 | 3 | - | 0 | 3 | 0 | 0 |  | 0 | 0 | 0 | 3 |
| Sawtooth | 2018 | 3 | 3 | 0 | 3 | 0 | 0 |  | 2 | 1 | 0 | 0 |
| Trout Creeks – east | 2016 | 12 | - | 0 | 12 | 0 | 0 |  | 0 | 11 | 0 | 1 |
| Trout Creeks – east | 2017 | 2 | - | 0 | 2 | 0 | 0 |  | 0 | 2 | 0 | 0 |
| Trout Creeks – east | 2018 | 1 | - | 0 | 1 | 0 | 0 |  | 0 | 1 | 0 | 0 |
| Trout Creeks – south | 2016 | 2 | - | 0 | 2 | 0 | 0 |  | 0 | 2 | 0 | 0 |
| Trout Creeks – south | 2017 | 1 | - | 0 | 1 | 0 | 0 |  | 0 | 1 | 0 | 0 |
| Trout Creeks – south | 2018 | 1 | - | 0 | 1 | 0 | 0 |  | 0 | 1 | 0 | 0 |
| Trout Creeks – west | 2016 | 2 | - | 0 | 2 | 0 | 0 |  | 0 | 2 | 0 | 0 |
| Trout Creeks – west | 2017 | 3 | - | 0 | 3 | 0 | 0 |  | 0 | 3 | 0 | 0 |
| Trout Creeks – west | 2018 | 1 | - | 0 | 1 | 0 | 0 |  | 0 | 1 | 0 | 0 |
| Three Forks | 2016 | 1 | - | 0 | 1 | 0 | 0 |  | 0 | 0 | 0 | 1 |
| Ten Mile | 2016 | 3 | - | 0 | 3 | 0 | 0 |  | 3 | 0 | 0 | 0 |
| Ten Mile | 2017 | 1 | - | 0 | 1 | 0 | 0 |  | 1 | 0 | 0 | 0 |
| Upper Owyhee | 2016 | 4 | - | 0 | 4 | 0 | 0 |  | 2 | 1 | 0 | 1 |
| **N** |  | 95 |  | 3 | 91 | 1 | 0 |  | 34 | 37 | 7 | 17 |

**Table S4.** Proportion of *Mycoplasma ovipneumoniae* exposed (exposed, indeterminate, and unexposed) and actively infected (positive (+), indeterminate, negative (-)) adult female bighorn sheep (*Ovis canadensis*), as determined by cELISA and PCR for populations (n = 13) in southeastern Oregon and northern Nevada. Pop. n = approximate number of adult females in each population, and n = number of individuals tested between 2016 and 2018.

|  | **Pop.** | ***M. ovipneumoniae* cELISA** | | | |  | ***M. ovipneumoniae* PCR prevalence** | | | |
| --- | --- | --- | --- | --- | --- | --- | --- | --- | --- | --- |
| **Population** | **n** | **n** | **Exposed** | **Indeterminate** | **Unexposed** |  | **n** | **+** | **Indeterminate** | **-** |
| Bowden Hills | 14 | 3 | 1.00 | 0.00 | 0.00 |  | 3 | 0.00 | 0.33 | 0.67 |
| Blue Mountain | 12 | 5 | 0.00 | 0.00 | 1.00 |  | 5 | 0.00 | 0.00 | 1.00 |
| Calicos | 25 | 5 | 0.60 | 0.20 | 0.20 |  | 5 | 0.00 | 0.00 | 1.00 |
| Eight Mile | 32 | 5 | 0.60 | 0.00 | 0.40 |  | 6 | 0.17 | 0.00 | 0.83 |
| Martin Creek | 14 | 5 | 0.80 | 0.00 | 0.20 |  | 6 | 0.00 | 0.00 | 1.00 |
| Rattlesnake | 53 | 21 | 0.62 | 0.29 | 0.10 |  | 21 | 0.05 | 0.00 | 0.95 |
| Sawtooth | 11 | 3 | 0.67 | 0.00 | 0.33 |  | 3 | 0.00 | 0.00 | 1.00 |
| Trout Creeks – east | 35 | 14 | 0.00 | 0.00 | 1.00 |  | 15 | 0.00 | 0.00 | 1.00 |
| Trout Creeks – south | 20 | 4 | 0.00 | 0.00 | 1.00 |  | 4 | 0.00 | 0.00 | 1.00 |
| Trout Creeks – west | 43 | 6 | 0.00 | 0.00 | 1.00 |  | 6 | 0.00 | 0.00 | 1.00 |
| Three Forks | 10 | 0 | - | - | - |  | 1 | 0.00 | 0.00 | 1.00 |
| Ten Mile | 17 | 4 | 1.00 | 0.00 | 0.00 |  | 4 | 0.00 | 0.00 | 1.00 |
| Upper Owyhee | 17 | 3 | 0.67 | 0.00 | 0.33 |  | 5 | 0.00 | 0.00 | 1.00 |

**Table S5.** Genetic diversity (observed heterozygosity, *H*_O_ averaged across 16 loci; expected heterozygosity, *H*_E_, averaged across 16 loci; allelic richness, *A*_R_, averaged across 16 loci), for populations of bighorn sheep (*Ovis canadensis*) in southeastern Oregon and northern Nevada.

| **Population** | **n** | ***H*_O_** | ***H*_E_** | ***A*_R_** |
| --- | --- | --- | --- | --- |
| Bowden Hills | 18 | 0.481 | 0.428 | 2.575 |
| Blue Mountain | 15 | 0.307 | 0.257 | 1.815 |
| Calicos | 17 | 0.360 | 0.350 | 2.410 |
| Eight Mile | 14 | 0.557 | 0.476 | 2.656 |
| Martin Creek | 12 | 0.372 | 0.363 | 2.501 |
| Rattlesnake | 26 | 0.496 | 0.447 | 2.678 |
| Sawtooth | 12 | 0.445 | 0.422 | 2.880 |
| Trout Creek – east | 20 | 0.345 | 0.316 | 1.994 |
| Trout Creek – south | 17 | 0.316 | 0.281 | 1.890 |
| Trout Creek – west | 18 | 0.350 | 0.329 | 2.017 |
| Ten Mile | 12 | 0.482 | 0.457 | 2.699 |
| Upper Owyhee | 10 | 0.356 | 0.334 | 2.375 |

**Table S6.** Hardy-Weinberg multi-population test results by locus. Results were generated using Markov chain parameters for all tests (dememorization = 1,000; batches = 100; iterations per batch = 1,000).

| **Locus** | **p-values** | **SE** |
| --- | --- | --- |
| AE129 | 0.406 | 0.00 |
| AE16 | 0.507 | 0.00 |
| BL4 | 0.756 | 0.00 |
| FCB11 | 0.899 | 0.00 |
| FCB266 | 0.944 | 0.00 |
| FCB304 | 0.924 | 0.00 |
| HH62 | 0.912 | 0.00 |
| JMP29 | 0.852 | 0.00 |
| MAF209 | 0.796 | 0.00 |
| MAF33 | 0.999 | 0.00 |
| MAF36 | 0.807 | 0.00 |
| MAF48 | 0.220 | 0.00 |
| MAF65 | 0.124 | 0.00 |
| OarFCB193 | 0.589 | 0.01 |
| TCRBV62 | 0.527 | 0.01 |
| TGLA387 | 0.933 | 0.00 |

**Table S7.** Hardy-Weinberg multi-population test results by population and subpopulation. Results were generated using Markov chain parameters for all tests (dememorization = 1,000; batches = 100; iterations per batch = 1,000).

| **Population** | **p-values** |
| --- | --- |
| Bowden Hills | 0.945 |
| Blue Mountain | 0.986 |
| Calicos | 0.481 |
| Eight Mile | 0.950 |
| Martin Creek | 0.430 |
| Rattlesnake | 0.991 |
| Sawtooth | 0.606 |
| Three Forks***** | 0.600 |
| Trout Creek east | 0.793 |
| Trout Creek south | 0.943 |
| Trout Creek west | 0.520 |
| Ten Mile | 0.675 |
| Upper Owyhee | 0.600 |

**Table S8.** Mean pre- and post-parturition NDVI values across 13 populations of bighorn sheep (*Ovis canadensis*) in southeastern Oregon and northern Nevada for the period 2016-2018. Mean NDVI values were generated using pre-processed data obtain from Earth Explorer, managed by USGS’s Earth Resource Observation Center. Superscripted numbers indicate rank, from lowest to highest, of NDVI, a proxy for forage quality. n/a indicates years where populations were not sampled.

|  | **2016** | |  | **2017** | |  | **2018** | |
| --- | --- | --- | --- | --- | --- | --- | --- | --- |
|  | $\bar{\boldsymbol{x}}$ **NDVI** | |  | $\bar{\boldsymbol{x}}$ **NDVI** | |  | $\bar{\boldsymbol{x}}$ **NDVI** | |
|  | **pre-** | **post-** |  | **pre-** | **post-** |  | **pre-** | **post-** |
| **Population** | **parturition** | |  | **parturition** | |  | **parturition** | |
| Bowden Hills | n/a | n/a |  | n/a | n/a |  | 0.27^6^ | 0.28^1^ |
| Blue Mountain | n/a | n/a |  | 0.16^1^ | 0.39^2^ |  | 0.22^3^ | 0.40^4^ |
| Calicos | n/a | n/a |  | n/a | n/a |  | 0.19^1^ | 0.42^8^ |
| Eight Mile | n/a | n/a |  | 0.31^10^ | 0.57^11^ |  | 0.32^11^ | 0.56^11^ |
| Martin Creek | n/a | n/a |  | 0.22^3^ | 0.40^3^ |  | 0.26^5^ | 0.41^7^ |
| Rattlesnake | 0.39^5^ | 0.44^4^ |  | 0.20^2^ | 0.37^1^ |  | 0.28^9^ | 0.32^2^ |
| Sawtooth | n/a | n/a |  | 0.30^9^ | 0.42^5^ |  | 0.27^6^ | 0.38^3^ |
| Trout Creeks –east | 0.31^1^ | 0.49^6^ |  | 0.23^4^ | 0.50^10^ |  | 0.21^2^ | 0.43^10^ |
| Trout Creeks – south | 0.32^2^ | 0.42^4^ |  | 0.29^8^ | 0.46^9^ |  | 0.25^4^ | 0.42^8^ |
| Trout Creeks – west | 0.33^3^ | 0.36^1^ |  | 0.24^7^ | 0.41^4^ |  | 0.27^6^ | 0.40^4^ |
| Three Forks***** | 0.47^6^ | 0.41^2^ |  | 0.23^4^ | 0.42^5^ |  | n/a | n/a |
| Ten Mile | 0.38^4^ | 0.44^5^ |  | 0.32^11^ | 0.43^8^ |  | 0.28^9^ | 0.40^4^ |
| Upper Owyhee | 0.47^6^ | 0.41^2^ |  | 0.23^4^ | 0.42^5^ |  | n/a | n/a |

*****The data for Upper Owyhee (UOP) was used as a proxy for Three Forks (TFK) due to insufficient seasonal GPS locations for the single collared adult female bighorn sheep in the TFK population. Both TFK and UOP occur within the upper part of the Owyhee Canyon.

**Table S9.** Correlation of fixed effects predicting survival of bighorn sheep (*Ovis canadensis*) lamb survival. Fixed effects include *Mycoplasma ovipneumoniae* status, *H*_E_ (expected heterozygosity), pre-NDVI (pre-parturition NDVI), and post-NDVI (post-parturition NDVI).

| **Fixed effects** | ***M. ovipneumoniae*** | ***H*_E_** | **pre-NDVI** | **post-NDVI** |
| --- | --- | --- | --- | --- |
| *M. ovipneumoniae* | - | 0.58 | -0.17 | -0.66 |
| *H*_E_ | 0.58 | - | 0.07 | -0.18 |
| pre-NDVI | -0.17 | 0.07 | - | 0.28 |
| post-NDVI | -0.66 | -0.18 | 0.28 | - |

**Table S10.** Breakdown of collared adult female bighorn sheep (*Ovis canadensis*) productivity by population (n = 13) and years (n = 3) in southeastern Oregon and northern Nevada. Data includes the number of adult females at the start of each year, the number of adult females pregnancy tested, and the proportion ($\hat{p}$) pregnant, the number of adult females dead prior to the parturition period, and the proportion of adult females observed with juveniles by year.

| **Population** | **year** | **# adult females at start of year** | **# adult females pregnancy tested** | $\hat{\boldsymbol{p}}$ **pregnant** | **# adult females dead pre-parturition** | $\hat{\boldsymbol{p}}$ **adult females observed with juveniles** |
| --- | --- | --- | --- | --- | --- | --- |
| Bowden Hills | 2018 | 3 | 3 | 1.00 | 0 | 1.00 |
| Blue Mountain | 2016 | 3 | 3 | 1.00 | 2 | 0.00 |
| Blue Mountain | 2017 | 3 | 2 | 1.00 | 0 | 0.67 |
| Blue Mountain | 2018 | 3 | 0 | n/a | 0 | 0.33 |
| Calicos | 2017 | 3 | 3 | 1.00 | 2 | 0.00 |
| Calicos | 2018 | 4 | 0 | n/a | 0 | 0.75 |
| Eight Mile | 2017 | 4 | 4 | 1.00 | 0 | 1.00 |
| Eight Mile | 2018 | 6 | 3 | 1.00 | 0 | 1.00 |
| Martin Creek | 2017 | 4 | 4 | 1.00 | 1 | 1.00 |
| Martin Creek | 2018 | 4 | 0 | n/a | 1 | 1.00 |
| Rattlesnake | 2016 | 10 | 10 | 1.00 | 1 | 0.78 |
| Rattlesnake | 2017 | 20 | 11 | 1.00 | 0 | 0.90 |
| Rattlesnake | 2018 | 18 | 0 | n/a | 0 | 0.83 |
| Sawtooth | 2017 | 3 | 3 | 1.00 | 0 | 1.00 |
| Sawtooth | 2018 | 3 | 3 | 1.00 | 0 | 1.00 |
| Trout Creeks – east | 2016 | 12 | 12 | 0.67 | 1 | 0.64 |
| Trout Creeks – east | 2017 | 11 | 2 | 1.00 | 0 | 0.64 |
| Trout Creeks – east | 2018 | 12 | 1 | 1.00 | 0 | 0.17 |
| Trout Creeks – south | 2016 | 2 | 2 | 0.50 | 0 | 0.50 |
| Trout Creeks – south | 2017 | 3 | 1 | 1.00 | 0 | 1.00 |
| Trout Creeks – south | 2018 | 4 | 1 | 1.00 | 0 | 0.75 |
| Trout Creeks – west | 2016 | 2 | 2 | 1.00 | 0 | 1.00 |
| Trout Creeks – west | 2017 | 5 | 3 | 1.00 | 3 | 1.00 |
| Trout Creeks – west | 2018 | 3 | 1 | 1.00 | 0 | 1.00 |
| Three Forks | 2016 | 1 | 1 | 0.00***** | 0 | 1.00 |
| Three Forks | 2017 | 1 | 0 | n/a | 0 | 1.00 |
| Ten Mile | 2016 | 3 | 3 | 1.00 | 0 | 1.00 |
| Ten Mile | 2017 | 4 | 1 | 1.00 | 1 | 0.67 |
| Ten Mile | 2018 | 3 | 0 | n/a | 0 | 1.00 |
| Upper Owyhee | 2016 | 5 | 4 | 1.00 | 0 | 1.00 |
| Upper Owyhee | 2017 | 4 | 0 | n/a | 0 | 1.00 |

*****Three Forks adult female although not pregnant on the pregnancy test was observed with a lamb

**Table S11.** Number of collared adult female bighorn sheep (*Ovis canadensis*) in each population (n) at parturition of juvenile bighorn sheep, with observation rate (%) of juveniles accompanying collared adult females across all observation intervals for the period 2016 to 2018 in bighorn sheep populations (n = 13) in southeastern Oregon and northern Nevada.

|  | **2016** | | | **2017** | | | **2018** | | |
| --- | --- | --- | --- | --- | --- | --- | --- | --- | --- |
| **Population** | **n** | **%** | **Range (%)** | **n** | **%** | **Range (%)** | **n** | **%** | **Range (%)** |
| Bowden Hills | n/a | n/a | n/a | n/a | n/a | n/a | 4 | 96 | 83–100 |
| Blue Mountain | n/a | n/a | n/a | 2 | 100 | 100 | 1 | 1.00 | 100 |
| Calicos | n/a | n/a | n/a | n/a | n/a | 100 | 3 | 1.00 | 100 |
| Eight Mile | n/a | n/a | n/a | 4 | 100 | 100 | 6 | 92 | 67–100 |
| Martin Creek | n/a | n/a | n/a | 3 | 100 | 100 | 5 | 100 | 1.00 |
| Rattlesnake | 7 | 95 | 75–100 | 18 | 100 | 100 | 15 | 98 | 71–100 |
| Sawtooth | n/a | n/a | n/a | 3 | 78 | 60–100 | 3 | 100 | 1.00 |
| Trout Creeks – east | 7 | 72 | 50–88 | 7 | 93 | 83–100 | 2 | 100 | 1.00 |
| Trout Creeks – south | 1 | 100 | 100 | 3 | 100 | 100 | 3 | 100 | 1.00 |
| Trout Creeks – west | 2 | 85 | 83–88 | 2 | 100 | 100 | 3 | 95 | 86–100 |
| Three Forks | 1 | 100 | 100 | 1 | 80 | 80 | n/a | n/a | n/a |
| Ten Mile | 3 | 100 | 100 | 2 | 100 | 100 | 3 | 72 | 67–83 |
| Upper Owyhee | 4 | 88 | 67–100 | 3 | 90 | 83–100 | n/a | n/a | n/a |
|  | 25 | 91 | 50–100 | 48 | 95 | 60–100 | 48 | 96 | 67–100 |

**Table S12.** Breakdown of juvenile bighorn sheep (*Ovis canadensis*) mortalities tested for *Mycoplasma ovipneumoniae* via polymerase chain reaction (PCR). Samples from juvenile mortalities were collected in the field between 2016 and 2018.

| **Mortality ID** | **Date collected** | **Population** | **Sex** | ***M. ovipneumoniae***  **status** |
| --- | --- | --- | --- | --- |
| RSP-01_16 | 26-May-2016 | Rattlesnake | male | positive |
| RSP-02_16 | 26-May-2016 | Rattlesnake | male | positive |
| RSP-03_16 | 31-May-2016 | Rattlesnake | male | positive |
| RSP-01_17 | 28-May-2017 | Rattlesnake | unknown | positive |
| RSP-02_17 | 28-May-2017 | Rattlesnake | female | positive |
| RSP-03_17 | 31-May-2017 | Rattlesnake | unknown | positive |
| RSP-04_17 | 5-Jun-2017 | Rattlesnake | male | positive |
| RSP-05_17 | 7-Jun-2017 | Rattlesnake | female | positive |
| RSP-06_17 | 7-Jun-2017 | Rattlesnake | male | positive |
| RSP-09_17 | 25-Jun-2017 | Rattlesnake | male | positive |
| RSP-10_17 | 25-Jun-2017 | Rattlesnake | female | positive |
| TCP-01_17 | 8-May-2017 | Trout Creeks – south | female | negative |
| BHP-01_18 | 16-Jul-2018 | Bowden Hills | female | positive |
| BSP-01_18 | 5-Jul-2018 | Blue Mountain | unknown | negative |
| RSP-01_18 | 16-Jun-2018 | Rattlesnake | female | positive |
| RSP-04_18 | 18-Jun-2018 | Rattlesnake | male | positive |
| RSP-06_18 | 18-Jun-2018 | Rattlesnake | unknown | positive |

**Table S13.** Strain-typed samples from bighorn sheep captured in southeastern Oregon and northern Nevada populations. All samples were multi-locus sequence typed (MLST) at the available sequences, 16S-23S intergenic spacer region (IGS), the small ribosomal subunit (16S), genes encoding RNA polymerase B (rpoB), and gyrase B (gyrB) were identical within loci. Details include year sample was collected, location of sample collection, WADDL processing number, animal details, and loci that were genotyped.

| **Date** | **Location** | **WADDL_#** | **Animal detail** | **IGS** | **16S** | **rpoB** | **gyrB** |
| --- | --- | --- | --- | --- | --- | --- | --- |
| 2004 | Sawtooth, Santa Rosa Range | 01370 | adult male | x |  |  |  |
| 2004 | Sawtooth, Santa Rosa Range | 01370 | adult male | x | x | x |  |
| 2012 | Snowstorm Mountains* | 12853 | adult female | x | x | x | x |
| 2014 | Santa Rosa Range | 00726 | unknown | x |  |  |  |
| 2014 | Santa Rosa Range | 00726 | unknown | x | x | x | x |
| 2015 | Santa Rosa Range | 04278 | unknown | x | x | x | x |
| 2016 | Rattlesnake | 00793 | adult female | x | x | x | x |
| 2018 | Bowden Hills | 12749 | juvenile female | x | x | x | x |
| 2018 | Rattlesnake | 12749 | juvenile male | x | x | x | x |
| 2019 | Rattlesnake | 16781 | unknown | x | x | x | x |

***** Individual dispersed from Santa Rosa Range and was re-captured in the Snowstorm Mountains

**Table S14.** Univariate models examining the effects of various *Mycoplasma ovipneumoniae* and genetic diversity covariates on juvenile bighorn sheep (*Ovis canadensis*) survival in southeastern Oregon and northern Nevada.

| **Model** | ***K*** | **AIC*_c_*** | **ΔAIC*_c_*** |
| --- | --- | --- | --- |
| *M. ovipneumoniae* (presence of infected juveniles) | 2 | 400.46 | - |
| *M. ovipneumoniae* (PCR) | 2 | 409.59 | 9.13 |
| *M. ovipneumoniae* (ELISA) | 2 | 422.65 | 22.19 |
| Expected heterozygosity (*H*_E_) | 1 | 417.59 | - |
| Allelic richness (*A*_R_) | 1 | 420.85 | 3.26 |

**Supplementary Figures**

**
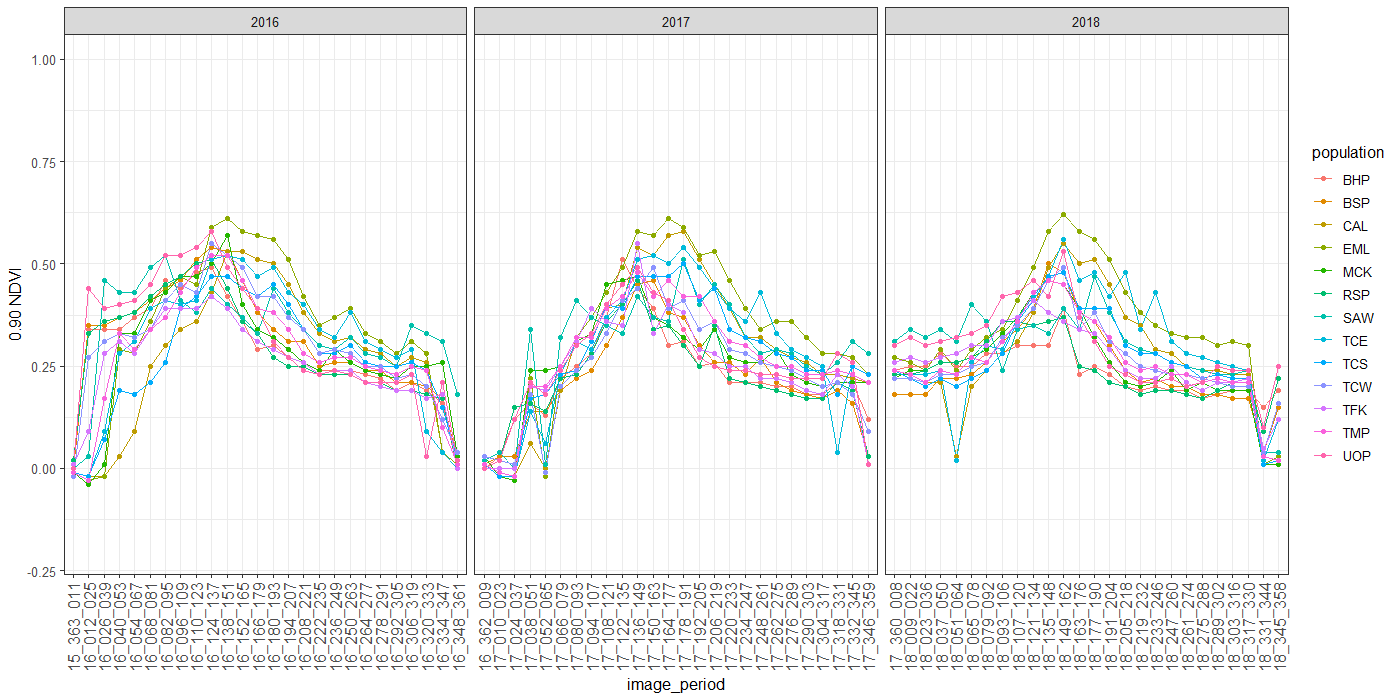
**

**Figure S1.** Ninetieth percentile normalized differential vegetation index (NDVI) values (scaled between -0.2 and 1.0) for all 13 bighorn sheep (*Ovis canadensis*) populations in southeastern Oregon and northern Nevada. Each value is derived from 14-day composite, 250 m resolution NDVI data from the Moderate Imaging Spectroradiometer (eMODIS) using annual composite 95% utilization distributions of collared adult females in each population to extract data. Population codes represent, BHP – Bowden Hills, BSP – Blue Mountain, CAL – Calicos, EMP – Eight Mile, MCK – Martin Creek, RSP – Rattlesnake, SAW – Sawtooth, TCE – Trout Creeks - east, TCS – Trout Creeks - south, TCW – Trout Creeks - west, TFK – Three Forks, TMP – Ten Mile and UOP - Upper Owyhee.
